# Expression profile analysis of the inflammatory response regulated by hepatocyte nuclear factor 4α

**DOI:** 10.1186/1471-2164-12-128

**Published:** 2011-02-25

**Authors:** Zhongyan Wang, Eric P Bishop, Peter A Burke

**Affiliations:** 1 Department of Surgery, Boston University School of Medicine, Boston, Massachusetts 02118, USA; 2 Bioinformatics Graduate Program, Boston University, Boston, Massachusetts 02215, USA

## Abstract

**Background:**

Hepatocyte nuclear factor 4α (HNF4α), a liver-specific transcription factor, plays a significant role in liver-specific functions. However, its functions are poorly understood in the regulation of the inflammatory response. In order to obtain a genomic view of HNF4α in this context, microarray analysis was used to probe the expression profile of an inflammatory response induced by cytokine stimulation in a model of HNF4α knock-down in HepG2 cells.

**Results:**

The expression of over five thousand genes in HepG2 cells is significantly changed with the dramatic reduction of HNF4α concentration compared to the cells with native levels of HNF4α. Over two thirds (71%) of genes that exhibit differential expression in response to cytokine treatment also reveal differential expression in response to HNF4α knock-down. In addition, we found that a number of HNF4α target genes may be indirectly mediated by an ETS-domain transcription factor ELK1, a nuclear target of mitogen-activated protein kinase (MAPK).

**Conclusion:**

The results indicate that HNF4α has an extensive impact on the regulation of a large number of the liver-specific genes. HNF4α may play a role in regulating the cytokine-induced inflammatory response. This study presents a novel function for HNF4α, acting not only as a global player in many cellular processes, but also as one of the components of inflammatory response in the liver.

## Background

Hepatocyte nuclear factor 4α (HNF4α) is a highly conserved member of the nuclear receptor superfamily. It is highly expressed in liver, kidney, intestine, and pancreas in mammals. The active form of HNF4α is a homodimer which recognizes a direct repeat (DR) of the AGGTCA motif separated by 1 nucleotide (DR1) as a binding site. HNF4α exerts direct transcriptional effects on target genes, and it also works indirectly *via *the positive regulation and negative regulation of other liver-enriched transcription factors, each of which regulates numerous downstream targets [1,2]. In contrast to the liver-enriched transcription factors HNF1α, HNF3α, HNF6, and CAAT/enhancer-binding protein (C/EBP)α, which when disrupted in the mouse genome the mice are viable but show specific effects on hepatocyte differentiation, metabolic function, and gene expression [3-6], disruption of the mouse *HNF4a *gene is embryonic lethal [7]. Studies with HNF4α deficient mice [8] have established the critical role of this factor in regulating diverse liver functions, including glucose, fatty acid and cholesterol homeostasis, bile acid and urea biosynthesis [9-11]. Defects in HNF4α function have been linked to the human disease maturity onset diabetes of the young 1(MODY1) that results from haploinsufficiency of *HNF4a *gene [12]. The pivotal role of HNF4α in the maintenance of the differentiated hepatic phenotype is highlighted by the exceptionally high number of potential target genes revealed by genome-scale target search studies. Binding sites for HNF4α in genes expressed in the liver occur more frequently than those of other liver-enriched transcription factors [13], supporting the idea that HNF4α is a global regulator of liver gene expression.

Diverse signaling pathways regulate the transcription of hepatocyte-specific genes. For example, trauma or infection results in the release of proinflammatory cytokines, *e.g.*, interleukin (IL)-6, IL-1, and tumor necrosis factor-α (TNF-α). The release of these cytokines has long been known to stimulate the acute phase response (APR) and rapidly alters rates of synthesis of a group of plasma proteins known as acute phase proteins (APPs) [14]. APPs are an established diagnostic tool as early indicators of inflammation and disease. Many APPs play beneficial roles in mediating the complex inflammatory response and seeking to restore homeostasis, but prolonged exposure to acute phase conditions has been correlated with inflammatory syndromes such as sepsis and multiple organ failure [15,16]. An understanding of the molecular events that are involved in mediating the response to external stresses can lead to the development of therapeutic strategies for preventing the progression of the APR to the chronic inflammatory states, while preserving its protective effects.

APP gene expression is regulated at the level of transcription. Transcriptional activation is mediated by a number of transcription factors. Beyond the well-known nuclear factor-κB, (NF-κB) and signal transducer and activator of transcription (STAT) family members, HNF4α has been shown to be involved in the regulation of liver-specific genes, including acute phase genes [17,18]. It has been reported that in several injury models, injury leads to significant changes in binding activities of several liver-enriched transcription factors, including HNF4α [18-20]. However, the functional analysis of HNF4α regulated-APR genes so far has mainly relied on the description of the expression level of a few selected genes [21]. To achieve a global view of HNF4α during the APR, we used microarray analysis to evaluate the expression profile in HepG2 cell, a human hepatoma cell line. This cell line is similar to hepatocytes in terms of biologic responsiveness [22-24] and is widely used as a model system for studying the regulation of acute phase protein synthesis in human liver [25-27]. In this study, HepG2 cells were treated with either HNF4α short hairpin RNA (shRNA) or cytokines (IL-6, IL-1β, TNF-α) alone, or in combination of the two treatments. Our results demonstrate that HNF4α is an important regulator in liver gene expression. The highly significant overlap of genes sensitive to HNF4α knock-down and cytokine treatment suggests that HNF4α may be involved in the regulation of the liver's inflammatory response. Our data also show that HNF4α may mediate a certain amount of genes indirectly *via *the ETS-domain transcription factor ELK1, a mitogen-activated protein kinase (MAPK)-responsive transcription factor [28,29], which is a previous undefined mechanism for HNF4α regulation.

## Results and discussion

### Knock-down endogenous HNF4α in HepG2 cells

**Figure 1:**
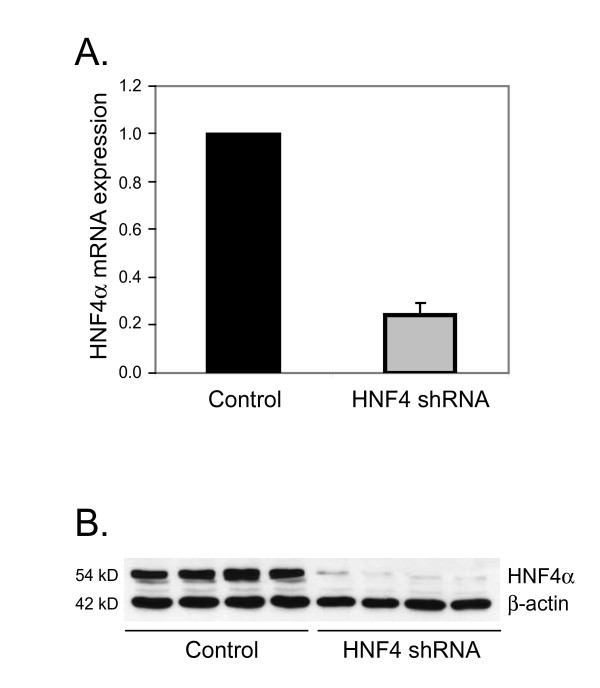
**Knock-down of HNF4α in HepG2 cells**. HepG2 cells were transfected with non-specific shRNA control or HNF4α shRNA plasmid. mRNA and whole cell lysates were prepared for real-time PCR (A) and Western blots (B), respectively. The results shown in (A) represent the relative mRNA expression level normalized to GAPDH mRNA level. The abundance of mRNA in the controls was set at 1. Data represent mean ± SD of 4 replicates. An HNF4α antibody (sc-6556, Santa Cruz Biotechnology) and β-actin (Sigma) antibody, used as an internal loading control, were utilized for Western blot (B).

To study the role of HNF4α in liver-specific gene expression and the inflammatory response, the endogenous HNF4α in HepG2 cells was knocked down by the technique of RNA interference. As shown in Figure 1, HNF4α shRNA caused a reduction in mRNA and protein levels of HNF4α by more than 70% relative to control levels assayed by real-time PCR and Western blot. Our results indicate that HNF4α shRNA can efficiently and specifically knock-down HNF4α in HepG2 cells [21].

### HNF4α acts as a global regulator of liver-specific gene expression

Given that HNF4α is a central mediator in hepatocyte-specific gene expression and liver function, it is important to identify the full spectrum of genes impacted by the loss of HNF4α. To reach this end, microarray analysis was performed to probe differences in gene expression between the control and HNF4α knock-down HepG2 cells. Because we are particularly interested in how HNF4α alters global gene expression patterns during the inflammatory response, we also examined gene expression in response to the inflammatory response induced by cytokine treatment.

**Figure 2:**
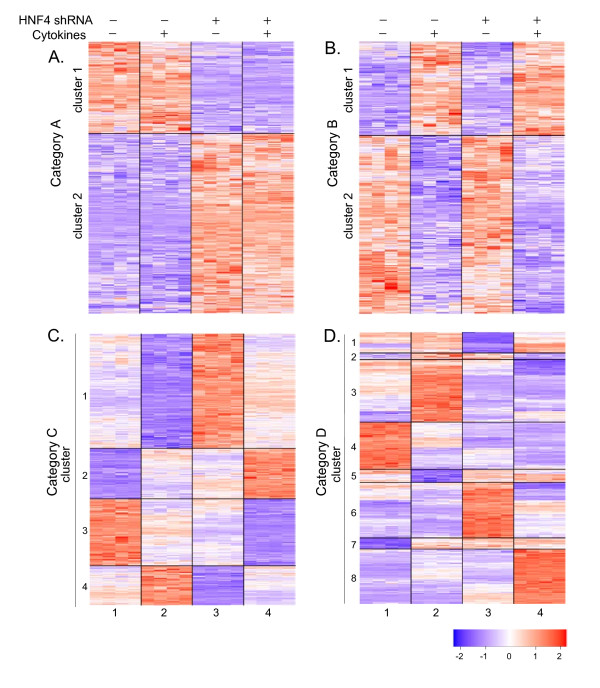
**The clustering expression profiles of up- and down-regulated genes**. A global transcriptional view of HepG2 cells in response to the treatment with cytokines and HNF4α knock-down alone (2 and 3) or in combination (4) is shown. Each group has 4 replicates. Relative expression values are expressed as a color code (bar color chart on the bottom, red up- and blue down-regulation). **(A)**, Category A contains genes that are significantly regulated by HNF4α knock-down, but not by cytokines; **(B)**, Category B contains genes that respond to cytokine treatment, but not to HNF4α knock-down; **(C)**, Category C contains genes whose expression is dependent on both treatments with cytokines and HNF4α knock-down in an independent manner; **(D)**, Category D has genes that exhibit an expression pattern dependent on both treatments in an interactive manner.

A two-way ANOVA was performed to identify differentially expressed genes and classify the observed expression patterns. This procedure identifies genes with altered expression in response to HNF4α shRNA or cytokines alone, or in combination HNF4α knock-down with cytokine treatments. The entire dataset is available at the NCBI Gene Expression Omnibus http://www.ncbi.nlm.nih.gov/geo/ with the accession number GSE15991. From this analysis, a total of 14,220 unique probesets were found to be present in the samples. Two-way ANOVA analysis was then used to determine which probesets were differentially expressed between the untreated control and treated groups. The ANOVA analysis identified four categories of interest (Figure 2): Category A, "HNF4α shRNA only" contains genes that are significantly regulated by HNF4α shRNA, but not by cytokines; Category B, "Cytokine only" contains genes that respond to cytokine treatment, but not to HNF4α knock-down; Category C, "Additive" contains genes whose expression is dependent on both treatments with cytokines and HNF4α shRNA, and the effect of both treatments is additive, suggesting that the two treatments likely influence expression independent of each other. Finally, Category D, "Interactive" contains genes that exhibit an expression pattern dependent on both treatments in which the effect of the two treatments is not additive, rather interactive between each other. The genes with significantly altered expression and fold changes in each category are listed in Additional file 1. Utilizing K-means clustering, the genes in the category A and category B (Figure 2A and 2B) were clustered into 2 clusters for each category; Category C and category D (Figure 2C and 2D) were clustered into 4 and 8 clusters, respectively.

As shown in Table 1, we identified a total of 5,173 probesets (36% of the 14,220 probesets on the Affymetrix HG U133A 2.0 GeneChip with sequence-specific signal in our experiment) that exhibited differential expression in response to HNF4α knock-down (false discovery rate, FDR< 0.01). Of these, 3,606 probesets were only affected by HNF4α knock-down, and were not differentially expressed in response to cytokine stimulation (Category A). The remaining 1,567 probesets exhibited a differential expression pattern that was either specific to the combined effect of HNF4α knock-down and cytokine treatments (502 probesets; Category D) or the genes that were affected by HNF4α knock-down and cytokine treatments independently (1,065 probesets; Category C). Among the probesets affected by HNF4α knock-down independently (Category A and Category C), many (3,088 of 4671 probesets) were up-regulated when HNF4α expression was reduced. These observations may suggest that HNF4α directly or indirectly regulates a large number of liver-specific gene expressions. We found that in our experimental conditions more genes appeared to be down-regulated or repressed under a normal level of HNF4α in HepG2 cells. This finding is not in agreement with the description that HNF4α functions primarily as a transcription activator [8,30], but is more in line with the observation that HNF4α can act as a suppressor of transcription [31,32]. To exclude an off-target effect of knock-down in our study, siRNA rescue experiments were performed. A rescue effect was observed in the knock-down HNF4α HepG2 cells transfected with an HNF4α siRNA-resistant construct generated by introducing silent substitutions in the HNF4α siRNA-target region (Additional files 2 and 3). These siRNA rescue experiments suggest that the HNF4α shRNA used in this study can specifically knock-down HNF4α in HepG2 cells.

**Table 1:** Genes exhibiting altered expression by the treatment with HNF4α shRNA alone and in combination with cytokines.

Cut-off ^1^	HNF4 shRNA only^2^	HNF4 shRNA+Cytokines^3^			Total
			
		Additive	Interactive	Total	
FDR < 0.01	3606	1065	502	1567	**5173**
2-fold	878	223	296	519	**1397**

Two methods of cut-off ^1^, FDR (false discovery rate) and 2-fold changes compared to untreated controls, are shown. HNF4 shRNA only^2 ^(Category A) denotes that gene expressions are significantly changed by HNF4α shRNA, but not by cytokines. HNF4 shRNA+Cytokines^3 ^indicate that gene expressions are significantly changed by both HNF4α shRNA and cytokines, including the additive (Category C) and interactive (Category D) effects.

The observation that a large number of liver specific genes are affected by HNF4α is consistent with the recent work of Odom *et al. *[13], in which the authors demonstrated that HNF4α binds the promoters of 12% of genes in human liver cells, whereas HNF1α binds the promoters of only 1.6% of genes using chromatin immunoprecipitation-chip (ChIP-chip). In order to determine what fraction of genes that showed differential expression in response to HNF4α knock-down is likely to be bound by HNF4α, we compared our microarray expression data to the ChIP-chip data reported by Odom *et al. *Only those genes in the Odom's data for which there were probesets on our microarray were considered. We found that 54% (659 of 1,219) of the genes bound by HNF4α detected by ChIP-chip also showed differential expression in response to HNF4α knock-down on our microarray. A Fisher-Exact test was used to test whether the fraction of genes in the Odom *et al. *data that are differentially expressed in response to HNF4α knock-down is significantly greater than the fraction of genes probed on our microarray that exhibit HNF4α-dependent expression, resulting in a highly significant *p*-value of *p *= 2.9 × 10^-33^. A similar analysis determined that 13% (659/5,173, *p *= 2.8 × 10^-17^) of those genes that exhibit differential expression in response to HNF4α knock-down were reported by Odom *et al. *to be bound by HNF4α. These results suggest that a substantial fraction of HNF4α-sensitive genes may be indirect targets of HNF4α. However, the differences seen might also be caused by the significant differences in the experimental methods used such as the data from ChIP-chip were derived from human hepatocytes, while our experiment was performed on HepG2 cells.

### Majority of genes affected by cytokines are also affected by HNF4α knock-down

As shown in Table 2, we found that expression of 15% of the probesets (2,202 of 14,220) was affected by treatment with cytokine alone (635 probesets; Category B), and treatment with both HNF4α shRNA and cytokines (1567 probesets; Category C and D). Of the probesets that are differentially expressed in response to cytokine treatment but non-interactively with HNF4α knock-down (Category B and C) almost twice as many are down-regulated (1,144 probesets) as up-regulated (556 probesets). This may reflect that during the APR, there is an important role for the down-regulation of specific genes in response to an inflammatory stimulation, although the up-regulation of APR genes has been more extensively studied than those that are down-regulated.

**Table 2:** Genes exhibiting altered expression by the treatment with cytokines alone and in combination with HNF4α shRNA.

Cut-off	Cytokine only^1^	HNF4 shRNA+Cytokines^2^			Total
			
		Additive	Interactive	Total	
FDR < 0.01	635	1065	502	1567	**2202**
2-fold	78	223	296	519	**597**

Cytokine only^1 ^(Category B) indicates that gene expressions are significantly changed by cytokines, but not by HNF4α shRNA. HNF4 shRNA+Cytokines^2 ^indicate that gene expressions are significantly changed by both HNF4α shRNA and cytokines, including the additive (Category C) and interactive (Category D) effects.

More interestingly, we found that majority of those genes that exhibit differential expressions in response to cytokine treatment also reveal differential expression in response to HNF4α knock-down. Of the 2,202 probesets found to be responsive to cytokine treatment, 1,567 (71%) of them also show altered expression in response to knock-down of HNF4α, while only 635 probesets are not influenced by HNF4α knock-down (Table 2). The *p*-value for this level of over-representation, calculated using a Fisher Exact test, is 1.8 × 10^-207^. Some of these probesets are altered independently by cytokine treatment and reduction in HNF4α levels (1,065 probesets; Category C), while others show a pattern of expression where the effect of cytokine treatment and reduction in HNF4α levels is interdependent (502 probesets; Category D). When more stringent criteria were used for filtering our data, and only those probesets in response to cytokine treatment and/or HNF4α knock-down that change expression by more than two fold were chosen, we found that an even greater percentage (519/597, 87%) of probesets regulated by cytokines are also regulated by HNF4α (Table 2). This over-representation suggests that HNF4α may play a significant role in orchestrating the inflammatory response in hepatic gene expression.

Although there is significant overlap between those probesets regulated by cytokines and those regulated by HNF4α knock-down (Category C and D), the expression patterns in response to these two treatments are varied. For some probesets the response to HNF4α knock-down are in the same direction as cytokine treatment (99 probesets, clusters 4 and 7 in the interactive category D, Figure 2D). This group of genes may represent a direct linkage of HNF4α with the injury response and will serve as interesting targets for further study of the complex role of HNF4α in the response to injury. For other genes the effect of each treatment is in opposition to each other (144 probesets, clusters 3 and 5 in Category D, Figure 2D). These diverse regulatory patterns observed suggest that the effects of HNF4α knock-down as that of cytokine treatment are pleiotropic in nature affecting transcription events at basic levels allowing individual gene responses to be highly variable but none the less altered.

Previous work by our lab has demonstrated that HNF4α binding activities are significantly reduced in a burn injury mouse model and a cytokine-induced APR cell culture model [18,21]. We have shown, utilizing the cell culture model, that the decrease in HNF4α binding activity also affects HNF4α's ability to transactivate target genes [21]. The injury induced decrease in HNF4α binding may affect cellular transcription by simply decreasing the amount of effective HNF4α available for binding. Our ability to efficiently decrease HNF4α concentration utilizing RNA interference technique may mimic this decrease in HNF4α binding ability isolating this aspect of HNF4α's role in the injury response.

### Genes annotated as participating in inflammatory response exhibit distinct expression patterns

In order to further explore the function of HNF4α in the inflammatory response, a set of 170 genes annotated as playing a key role in inflammatory response was obtained from Gene ontology (GO) http://geneontology.org. These inflammatory response genes are highly enriched in the set of probesets up-regulated by cytokines (*p *= 3.5 × 10^-3^, 334% above background), but not in those probesets down-regulated after cytokine treatment. Slightly more inflammatory response genes are up-regulated in response to HNF4α knock-down than those that are down-regulated, but the difference is not statistically significant (*p *> 0.05).

**Figure 3:**
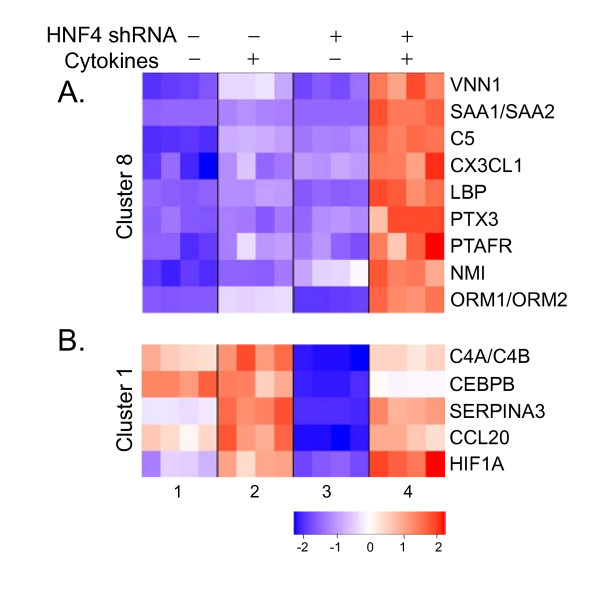
**Inflammatory response genes are enriched in two clusters within the interactive category (Category D)**. Expression profiles of up- and down-regulated genes in the different groups treated by cytokines and HNF4α knock-down alone (2 and 3) or in combination (4) are shown. Each group has 4 replicates. Relative expression values are expressed as a color code (bar color chart on the bottom, red up- and blue down-regulation). Inflammatory response genes (listed at the right side of the graph) extracted from GO are highly enriched in two different clusters (A and B). Gene abbreviations: VNN1, vanin 1; SAA1/SAA2, serum amyloid A1/serum amyloid A2; C5; complement component 5; CX3CL1, chemokine (C-X3-C motif) ligand 1; LBP, lipopolysaccharide binding protein; PTX3, pentraxin-related gene, rapidly induced by IL-1 beta; PTAFR, platelet-activating factor receptor; NMI, N-myc (and STAT) interactor; ORM1/ORM2, orosomucoid 1/orosomucoid 2; C4A/C4B, complement component 4A/complement component 4B; CEBPB, CCAAT/enhancer binding protein (C/EBP), beta; SERPINA3, serpin peptidase inhibitor, clade A (alpha-1 antiproteinase, antitrypsin), member 3; CCL20, chemokine (C-C motif) ligand 20; HIF1A, hypoxia-inducible factor 1, alpha subunit (basic helix-loop-helix transcription factor).

To determine whether certain expression patterns are associated with functional annotations, we further tested each cluster in each category using ANOVA analysis. While HNF4α-regulated probesets as a whole are not significantly enriched for the annotated inflammatory response genes, two expression clusters in the interactive category D are highly enriched for these genes. Of the 5,173 probesets regulated by HNF4α knock-down, 25 of them are annotated as participating in inflammatory response, and 14 of these genes fall into one of two clusters. One of these clusters (cluster 8, Category D) contains 101 probesets that are dramatically up-regulated by the combination of HNF4α shRNA and cytokine treatment, but exhibit relatively low levels of expression in the untreated controls, and the cells treated by either HNF4α knock-down or cytokines alone. Nine of 101 probesets in cluster 8 are annotated in GO as participating in the inflammatory response, which is 8.2 times more than expected by chance (*p *= 2.2 × 10^-6^) (Figure 3A). Another cluster (cluster 1, Category D) contains 41 probesets expressed in moderate levels under all conditions except HNF4α shRNA treatment in the absence of cytokines, in which they are markedly down-regulated. In this cluster, five genes are annotated as inflammatory response genes which is over 11.3 times more than expected by chance (*p *= 8.8 × 10^-5^) (Figure 3B). While it is not immediately clear why genes that play a role in inflammatory response are enriched in these particular expression clusters, it is intriguing that such genes exhibit similar HNF4α-dependent expression. These inflammatory response genes in cluster 8 and cluster 1 present good candidates for further study.

**Figure 4:**
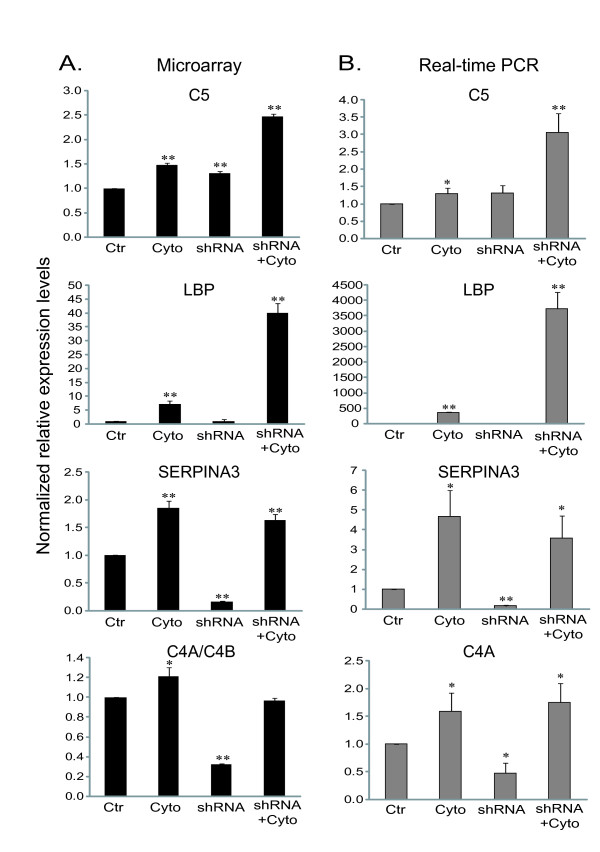
**Confirmation of microarray results using quantitative real-time PCR**. (**A**), Raw intensity values were measured using microarray. (**B**), Real-time PCR results for the same genes are expressed as the relative mRNA expression level normalized by GAPDH mRNA level. The abundance of mRNA in the controls was set at 1. Data represent mean ± SD of 4 replicates. Ctr, Control; Cyto, Cytokines; shRNA, HNF4α shRNA; shRNA+Cyto, HNF4α shRNA plus Cytokines. *p < 0.05 and **p < 0.01 indicate a significant difference compared to control. Gene abbreviations are the same as described in Figure 3 legend.

To confirm the microarray results, we chose several transcripts from category D, cluster 8 (C5 and LBP) and cluster 1 (SERPINA 3 and C4A), and measured their expressions with real-time PCR using the same RNA samples used for microarray studies. All of them showed a high concordance between microarray and real-time PCR data. Figure 4 illustrates the comparison of the expression levels between microarray (Figure 4A) and real-time PCR (Figure 4B) for the selected transcripts from cluster 8 and cluster 1 (Category D).

**Figure 5:**
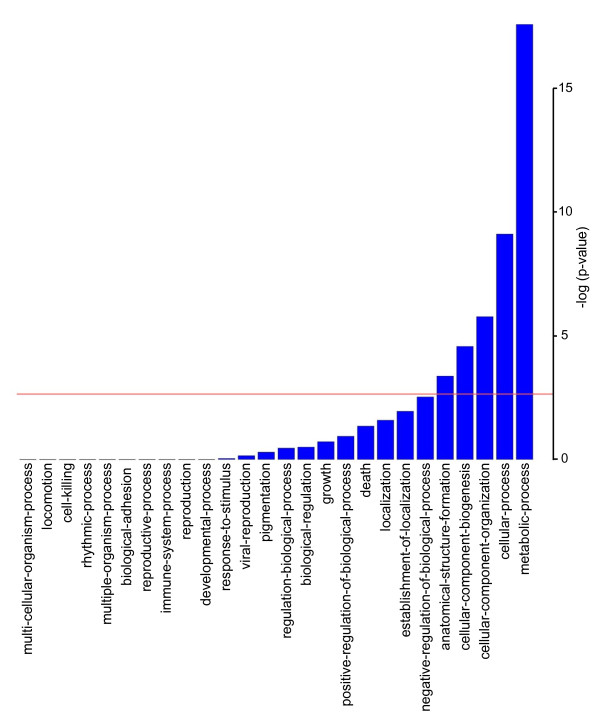
**Gene ontology analysis**. Twenty six broad categories of biological processes were analyzed for genes that exhibit HNF4α-dependent expression. Significance levels are plotted as-log (*p *value). One-tail *p*-values were calculated using the Fisher Exact test. Threshold (line) denotes the *p *= 0.002 level, which is the threshold for significance after Bonferroni correction.

In addition to testing for enrichment of inflammatory response genes, enrichment for broad categories of functionality was calculated for each cluster. GO terms of all 26 top level biological processes were analyzed. We found that GO term of metabolism is significantly enriched in genes exhibiting HNF4α-dependent expression (Figure 5). As previous work [9,30,33] has noted that HNF4α plays a significant role in regulating metabolism in the liver, it is not surprising that the GO term for metabolism is HNF4α-dependent. Thirty percent of genes (1,530 of 5,173) that are differentially expressed in response to HNF4α knock-down are annotated as participating in a metabolic process, resulting in a Fisher Exact *p*-value of 2.6 × 10^-18^.

### HNF4α may regulate a large number of targets *via *the ETS family of transcription factors

To gain insight into the complex transcriptional networks that regulate hepatic gene expression, the promoter region (-1 kb to +0.5 kb relative to the transcription start site, TSS) of genes was extracted, and potential transcription factor binding sites were identified using Clover [34]. By extensive search of the genes found to be differentially expressed as a function of HNF4α knock-down, we found that the HNF4α motif is not statistically enriched in the promoters of genes whose expression changes in response to HNF4α knock-down relative to promoters chosen randomly from throughout the genome (*p *> 0.05). There are several possible reasons for this observation. Firstly, HNF4α binding sites may be located far up-stream or down-stream of the TSS, although the results still remain the same when we extended our search up to 5 kb up-stream and 2.5 kb down-stream of the TSS. Secondly, there might be a fraction of sites that bind HNF4α but differ from the established consensus. Thirdly, HNF4α may interact with proximal promoters through formation of enhancer/promoter loops with HNF4α binding to distal regulatory element [35]. Yet another possibility is that HNF4α might act as a cofactor, interacting with other transcription factors and not directly bind to DNA.

**Figure 6:**
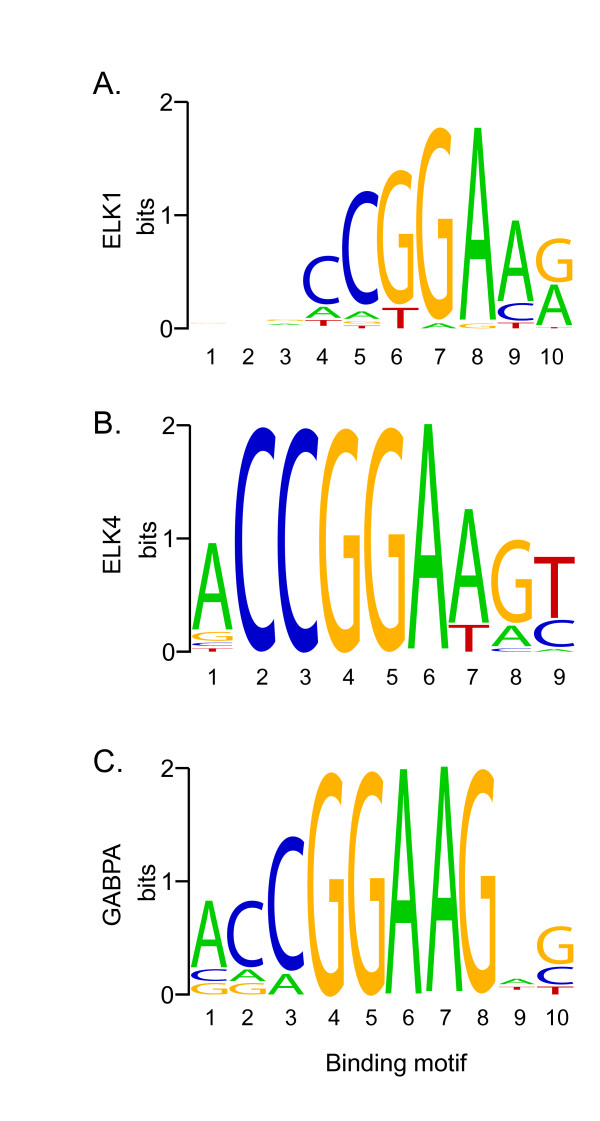
**Sequence logos of ETS transcription factor binding sites**. Sequence logos of consensus DNA binding sites for the three ETS transcription factors enriched in genes regulated by HNF4α. Y axis indicates amount of information at each position in the motif. These logos were generated from information obtained from the JASPAR core database [42]. **(A)**, ELK1 binding motif; **(B)**, ELK4 binding motif; and **(C)**, GABPA binding motif.

Indeed, we found that the binding sites for the ETS family of transcription factors including ELK1, ELK4 and GA binding protein transcription factor A (GABPA) are highly enriched in the HNF4α knock-down group. The binding motifs for ELK1 (*p *= 4.0 × 10^-7^), ELK4 (*p *= 1.14 × 10^-10^) and GABPA (*p *= 3.4 × 10^-5^) are very similar (Figure 6), and all significantly enriched in the promoter regions of genes affected by HNF4α knock-down. This mirrors a finding by Smith *et al. *[36], where they showed that ELK1 binding motifs are enriched in the promoters of genes also bound by HNF4α. Moreover, in this study we found that ELK1 and ELK4 genes exhibit significant differential expression in response to HNF4α knock-down. ELK1 is up-regulated when HNF4α is knocked down, while ELK4 is down-regulated. These results suggest that HNF4α may regulate a substantial number of genes *via *ELK transcription factors.

The transcription factor ELK1 is of particular interest as this protein is a nuclear target of extracellular-regulated kinases (ERKs) and plays a pivotal role in immediate early gene induction by external stimuli [28,29]. It has been known that HNF4α expression is modulated by MAP kinase signaling [37,38]. Li *et al. *[39] reported that ELK1 is involved in the inflammatory response *via *stimulation of chemokine by thrombin. These observations suggest that the relationship between ELK1 and HNF4α may be especially relevant to understanding the role of HNF4α in regulating the inflammatory response.

**Figure 7:**
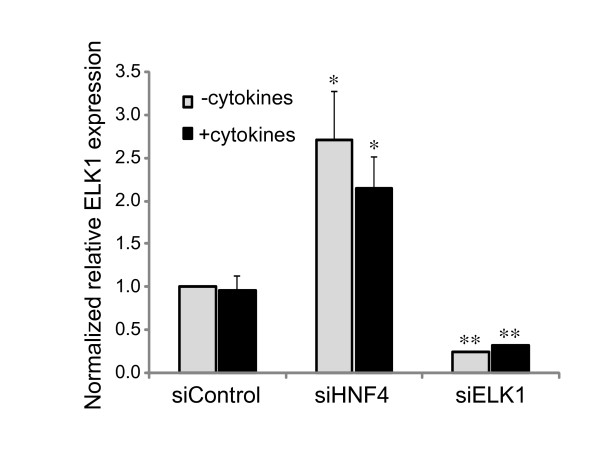
**The ELK1 expression in HepG2 cells with HNF4α or ELK1 knock-down**. HepG2 cells were transfected with non-specific siRNA control (siControl), HNF4α shRNA plasmid (siHNF4) or siELK1, and then treated with or without cytokines. The expression of ELK1 was measured by real-time PCR. The results represent the relative mRNA expression level normalized to GAPDH mRNA level. The abundance of mRNA in the controls was set at 1. Data represent mean ± SD of three different experiments. *p < 0.05 and **p < 0.01 indicate a significant difference compared to siControls.

**Figure 8:**
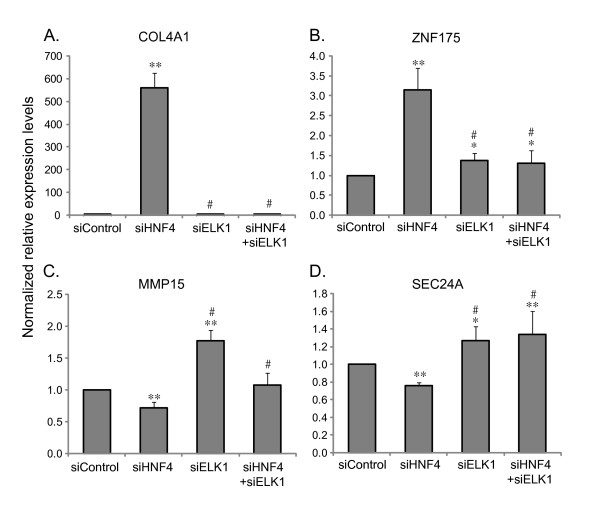
**A decrease in ELK1 expression leads to elimination of the regulatory effect of HNF4α knock-down on a subset of genes**. HepG2 cells were treated with non-specific siRNA control (siControl), HNF4α shRNA plasmid (siHNF4), siELK1 or both siHNF4 and siELK1. The expressions of COL4A1 (collagen, type IV, alpha 1), ZNF175 (zinc finger protein 175), MMP15 (matrix metallopeptidase 15) and SEC24A (SEC24 family, member A) genes were determined by real-time PCR. The abundance of mRNA in the siControl was set at 1. Data are presented as mean ± SD of three different experiments. *p < 0.05 and **p < 0.01 indicate a significant difference compared to siControl. #*p *< 0.01 indicates a significant difference compared to siHNF4α.

**Figure 9:**
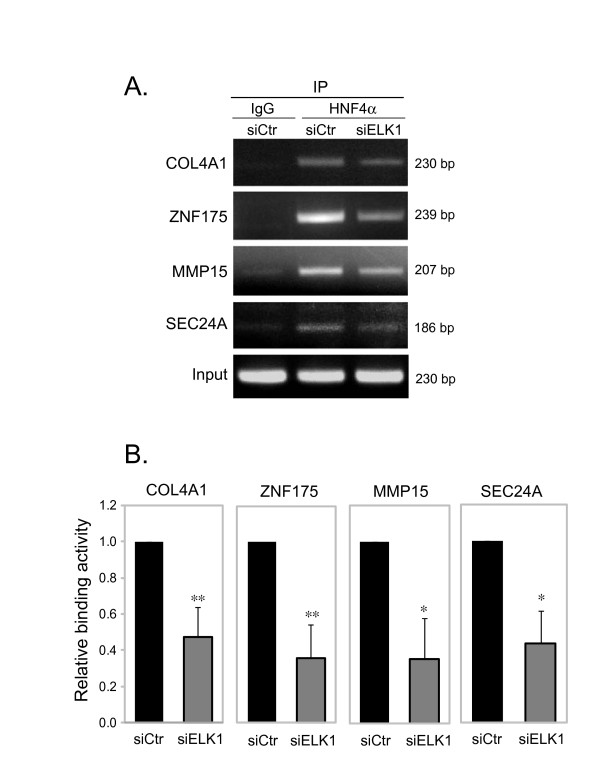
**The reduction of ELK1 level decreases the ability of HNF4α to interact with the promoters of a subset of genes**. **(A)**, HepG2 cells were transfected with siControl (siCtr) or siELK1. Protein interaction of HNF4α and ELK1 was determined by ChIP assay with either antibody against HNF4α or goat normal IgG (IgG). Chromatin-immunoprecipitated DNA was analyzed by PCR with primers specific for the ELK1 binding sites in the promoter regions of COL4A1, ZNF175, MMP15 and SEC24A genes. The result shown in **(A) **is a representative experiment, replicated three times with similar results. **(B)**, Histograms show densitometric analyses of relative binding abilities. Values represent mean ± SD of three separate experiments. The relative quantitative analysis was carried out by comparison of siELK1 with siControl, and the siControl was set at 1. *p < 0.05 and **p < 0.01 indicate a significant difference compared to siCtr. IP, immunoprecipitation.

By searching for ELK1 binding site in the HNF4α regulated genes (5173 genes), we identified 373 genes that have one or more potential ELK1 binding sites in their promoter regions (-1 kb to +0.5 kb) (Additional file 4). To define a functional link between HNF4α and ELK1, we selected 4 genes (COL4A1, ZNF175, MMP15 and SEC24A). These genes all were found having an ELK1 binding motif (CCGGAAG/A, Figure 6), but no HNF4α binding motif in their proximal promoter regions, and from our microarray analysis their expressions were shown to be either up-regulated (COL4A1, ZNF175) or down-regulated (MMP15 and SEC24A) by HNF4α knock-down. The expression of these potential ELK1 target genes was examined by real-time PCR in HepG2 cells treated with siRNA to knock-down HNF4α and/or ELK1 alone or both together. As shown in Figure 7, the expression of ELK1 can be efficiently knocked-down by siRNA. The knock-down of HNF4α resulted in a significantly greater ELK1 expression compared to control (*p *< 0.05), and the ELK1 expression was not significantly affected by cytokine treatment, which are consistent with our microarray data. We propose that HNF4α may indirectly regulate a number of genes through ELK1 transcription factor based on the observation that the ELK1 binding site is highly enriched in genes affected by HNF4α knock-down. It was predicted that if a higher ELK1 expression induced by HNF4α knock-down affects the transcriptional outcomes of ELK1-target genes, we should see an opposite regulatory effect on these gene transcriptions when the ELK1 level is reduced. The results (Figure 8) show that when the cells were treated with siELK1 either alone or with siHNF4α, the two genes up-regulated by HNF4α knock-down (Figures 8A and 8B) are expressed at significantly lower levels compared to HNF4α knocked-down (*p *< 0.01). In contrast, the genes down-regulated by HNF4α knock-down (Figures 8C and 8D) show significantly increased expression compared to siHNF4α (*p *< 0.01). This result is consistent with a direct effect of ELK1 level induced by the decrease of HNF4α concentration on a subset of ELK1-target genes. However, these findings could be explained by another mechanism by which an HNF4α/ELK1 complex is formed, leading to alterations in the regulation of a group of genes. To test this hypothesis, chromatin immunoprecipitation (ChIP) assays were performed. An HNF4α antibody was used to immunoprecipitate chromatin from HepG2 cells. Specific PCR primers were utilized to amplify a DNA fragment with an ELK1 binding motif, and without an HNF4α binding motif. As shown in Figure 9, HNF4α was able to interact with these genes containing ELK1 binding sites, and the interactive ability of HNF4α was significantly reduced after the knock-down of ELK1. Interestingly, when the HNF4α binding motif was further searched using a web-based search tool, HNF4 Motif Finder [40], a potential HNF4 binding site was identified in the promoter region of MMP15, which could lead to a direct binding of HNF4α when HNF4α antibody was used to immunoprecipitate chromatin. However, if HNF4α binding to MMP15 is independent of ELK1, when ELK1 is knocked down, one would expect to see no change in the binding of MMP15 in the ChIP assay, this is not the case as shown in Figure 9. Furthermore, we found that ELK1 can directly bind to its potential ELK1 binding site in MMP15 utilizing electrophoretic mobility shift analysis (data not shown). Given these findings, we hypothesize that HNF4α may indirectly mediate gene expression, in part, through a co-operative interaction with ELK1, and possibly also with other ETS transcription factors. Others have reported that a number of ETS family proteins interact and crosstalk with several transcription factors including AP-1, NF-κB and Stat-5 to co-regulate the expression of cell-type specific genes. Such interactions coordinate cellular processes in response to diverse signals including cytokines, growth factors, antigen and cellular stresses [41], here we believe that we have uncovered a novel interplay between the transcription factors HNF4α and ELK1 for controlling gene expression in the liver.

## Conclusions

HNF4α is a major regulator of hepatic gene expression. The complex physiological effects of HNF4α on the regulation and maintenance of hepatic phenotype are likely involved both directly and indirectly in a systemic response such as that seen in a response to injury or inflammation. Our microarray data are consistent with a broad effect of HNF4α on liver functions, and more importantly, the microarray analysis provides a genomic view for the role of HNF4α in the inflammation response, which greatly extends the observations from both animal- and cell culture-injury models. However, more experimentation and a focus on individual pathways will need to be done before a full picture of HNF-4α's role in the modulation of hepatic phenotype by injury can be obtained.

## Methods

### Cell culture and treatments

HepG2 cells (ATCC # HB-8065), human hepatoma cells, were grown in Dulbecco Modified Eagle Medium (DMEM) supplemented with penicillin (100 units/ml), streptomycin (100 μg/ml), and 10% heat-inactivated fetal bovine serum (Mediatech, Herndon, VA) at 37°C in a humidified atmosphere with 5% CO_2_.

The inflammatory response in HepG2 cells was stimulated with a cytokine mixture consisting of 1 ng/ml of recombinant human IL-1β, 10 ng/ml of IL-6, and 10 ng/ml of TNF-α (PeproTech, Rocky Hill, NJ) in serum-free medium for 18 h.

Knock-down of HNF4α in HepG2 cells was carried out as described previously [21]. Briefly, shRNA plasmids (HNF4α shRNA and non-specific control shRNA) were transfected into HepG2 cells with the Nucleofector (Amaxa Biosystem, Cologne, Germany) T-28 protocol following the manufacturer's instructions. Knock-down of ELK1 in HepG2 cells was performed by transfection with small interfering RNA (siRNA) (Ambion, Austin, TX) using lipofectamine 2000 reagent (Invitrogen, Carlsbad, CA) according to the manufacturer's instructions. Twenty-four hours after transfection, the cells were placed in serum-free medium for 5 h and then either treated or untreated with cytokines for 18 h.

The different treated HepG2 cells were divided into four groups: Group 1, control: the cells were transfected with non-specific control shRNA; Group 2, HNF4α shRNA: the cells were transfected with HNF4α shRNA; Group 3, cytokines: the control cells were transfected with non-specific control shRNA and then treated with cytokines; Group 4, HNF4α shRNA with cytokine treatment: the cells were transfected with HNF4α shRNA prior to the treatment with cytokines. Four biological replicates from separate experiments were performed for each of the four study groups.

### Total RNA isolation and real-time PCR

Total RNA was extracted using the RNeasy Mini Kit (Qiagen, Valencia, CA) according to the manufacturer's instructions. Quantitative real-time PCR analysis was conducted on the ABI 7000 Sequence Detection System and StepOnePlus™ Real-Time PCR System. Relative mRNA expression was quantified using the comparative Ct (ΔΔCt) method according to the ABI manual (Applied Biosystems, Foster City, CA). Amplification of glyceraldehyde-3-phosphate dehydrogenase (GAPDH) was used in each reaction as an internal reference gene. Assays were performed in triplicate. TaqMan probes were used for the human HNF4α (Hs00230853_m1), complement component 5 (C5, Hs00156197_m1), lipopolysaccharide binding protein (LBP, Hs00188074_m1), serpin peptidase inhibitor member 3 (SERPINA3, Hs00153674_m1), complement component 4A (C4A, Hs00167147_m1), ELK1 (Hs00901847_m1), collagen, type IV, alpha 1 (COL4A1, Hs01007469_m1), zinc finger protein 175 (ZNF175, Hs00232535_m1), matrix metallopeptidase 15 (MMP15, Hs00233997_m1), SEC24 family, member A (SEC24A, Hs00405228_m1) and GAPDH (Hs99999905_m1) from the TaqMan^®^Gene Expression Assays (Applied Biosystems).

### Microarray analysis

Affymetrix HG-U133A 2.0 GeneChips were used to study the expression levels for HepG2 cell RNA. Sample labeling, hybridization to microarrays, scanning and calculation of normalized expression levels were carried out at the Microarray Resource, Boston University School of Medicine, Boston, MA. RNA samples (four biological replicates for each of the four study groups: control, HNF4α shRNA, cytokines, and HNF4α shRNA with cytokine treatment) were analyzed. Microarray data were quantified and normalized using Affymetrix MicroArray Suite (MAS) 5.0. Two-way ANOVA analysis was then used to determine which probesets were differentially expressed between the untreated control and treated groups. Only probesets exhibiting differential expression with FDR < 0.01 were included in this study. After the ANOVA analysis was performed, K-means clustering was then used to cluster probesets that exhibit similar expression patterns within each category of differential expression. In order to eliminate multiple probesets, the raw data were clustered based on Unigene identifiers (ids), which represent unique transcription loci. Unigene ids associated with multiple probesets that exhibit different patterns of expression were removed from the dataset, ensuring unambiguous data that could be easily used in subsequent enrichment analyses. This approach ensures that, if anything, the results of subsequent analyses are conservative.

### Gene ontology (GO) enrichment analysis

Before GO enrichment could be calculated, each GO annotation was mapped to a corresponding Unigene id. SwissProt ids associated with each Unigene id were obtained and were used for this purpose. Where SwissProt ids were not available, gene symbols were used to associate GO annotations with Unigene ids. Each cluster in all 4 categories was then tested for enrichment for genes associated with inflammatory response GO database (relative to the entire set of genes on the microarray) using Fisher's Exact test. In addition, to identify other functionality, each cluster was tested for enrichment of each of the top-level "biological process" annotations in the GO database. As there are 26 top-level "biological process", a Bonferroni correction was applied for this top-level analysis, resulting in a *p*-value cut-off of 0.002.

### Promoter analysis

Weight matrices obtained from the JASPAR core database [42] were used to identify potential transcription factor binding sites in the up-stream of promoter regions. One promoter with each Unigene id was included in the analysis, so that genes with multiple annotated promoters/TSS did not bias the analysis. The region, extending from -1 kb to +0.5 kb relative to the TSS, was extracted. Clover [34] was used with a minimum log-ratio score cut-off of 8.0 to identify potential binding sites in this region. Promoters were classified based on whether they contain at least one binding site, and enrichment in different expression clusters relative to a background set of promoters derived from all probesets present on the microarray. The randomization feature of Clover was not used, instead the exact number of promoters containing binding sites in both the HNF4α-regulated set and the background set were computed, and a *p*-value was calculated using Fisher's Exact test. Because there are almost 140 transcription factors in the JASPAR database, a Bonferroni correction was applied, resulting in a *p*-value cut-off of 3.5 × 10^-4^.

### Chromatin immunoprecipitation (ChIP) assay

ChIP assay was performed as described previously [21]. The purified chromatin was immunoprecipitated using 10 μg of anti-HNF-4α (sc-6556, Santa Cruz Biotechnology, Santa Cruz, CA) or normal goat IgG (Santa Cruz). After DNA purification, the presence of the ELK1 putative binding motif (CCGGAAG/A) DNA sequence was assessed by PCR. The primers used were as follows: (1) COL4A1 gene: 5'-GAGTTTAGCGCAGGATGAGG-3' and 5'-GCTCTCCTGCTTGGGAGTAG-3', and the PCR product is 230 bp in length; (2) ZNF175: 5'-TAAAAGCCCTTTGACGATGG-3' and 5'-CTCTAGGCCACTTCCGGTTT-3', and the PCR product is 239 bp in length; (3) MMP15: 5'-ATCCAGCTCGTTAAGCTTCG-3' and 5'-TTAATCTCTCCGAGCCTCCA-3' for amplifying a 207 bp DNA fragment. (4) SEC24A: 5'-GCACCAGGAGCTGTCAGG-3' and 5'-GGCAGCCAAACCTAGAGAGA-3', and the PCR product is 186 bp in length. The PCR conditions were as following: 95°C for 10 min, followed by 94°C for 45 s, 58°C for 60 s, and 72°C for 60 s for a total of 31 cycles. In the ELK1 knock-down experiments, the relative quantitative analysis in binding activity was performed, utilizing densitometry and statistical analysis, by comparison of siELK1 with non-specific siRNA control, and the control was set at 1.

## Authors' contributions

ZW and PAB designed the experiment. ZW performed the experiments. EPB analyzed the data. ZW and EPB wrote the manuscript. All authors read and approved the final manuscript.

## Supplementary Material

Additional file 1 **Genes that respond to the treatment of HNF4α knock-down and/or cytokines**. This file includes four sub-tables showing the genes with significantly altered expression and fold changes in four categories (Category A to D). Click here for file

Additional file 2 **Construction of HNF4α siRNA-resistant mutant**. This file illustrates the generation and DNA sequencing of the HNF4α siRNA-resistant construct. Click here for file

Additional file 3 **siRNA rescue assay**. This file shows the characterization of the HNF4α siRNA-resistant construct by Western blot, and the rescue effect of this construct on HNF4α knock-down responsive genes. Click here for file

Additional file 4 **ELK1 and HNF4α binding motifs in HNF4α-regulated genes**. This file shows the sequence and position of ELK1 and HNF4α potential binding sites in the group of HNF4α-regulated genes. Click here for file
