# Supplementary material for: Expression profile analysis of the inflammatory response regulated by hepatocyte nuclear factor 4α
Source: BMC Genomics. 2011 Feb 25;12:128. doi: 10.1186/1471-2164-12-128 (PMC3053261; doi:10.1186/1471-2164-12-128)
Supplement: Additional file 2 — Construction of HNF4α siRNA-resistant mutant. This file illustrates the generation and DNA sequencing of the HNF4α siRNA-resistant construct. [file 1471-2164-12-128-S2.PDF]

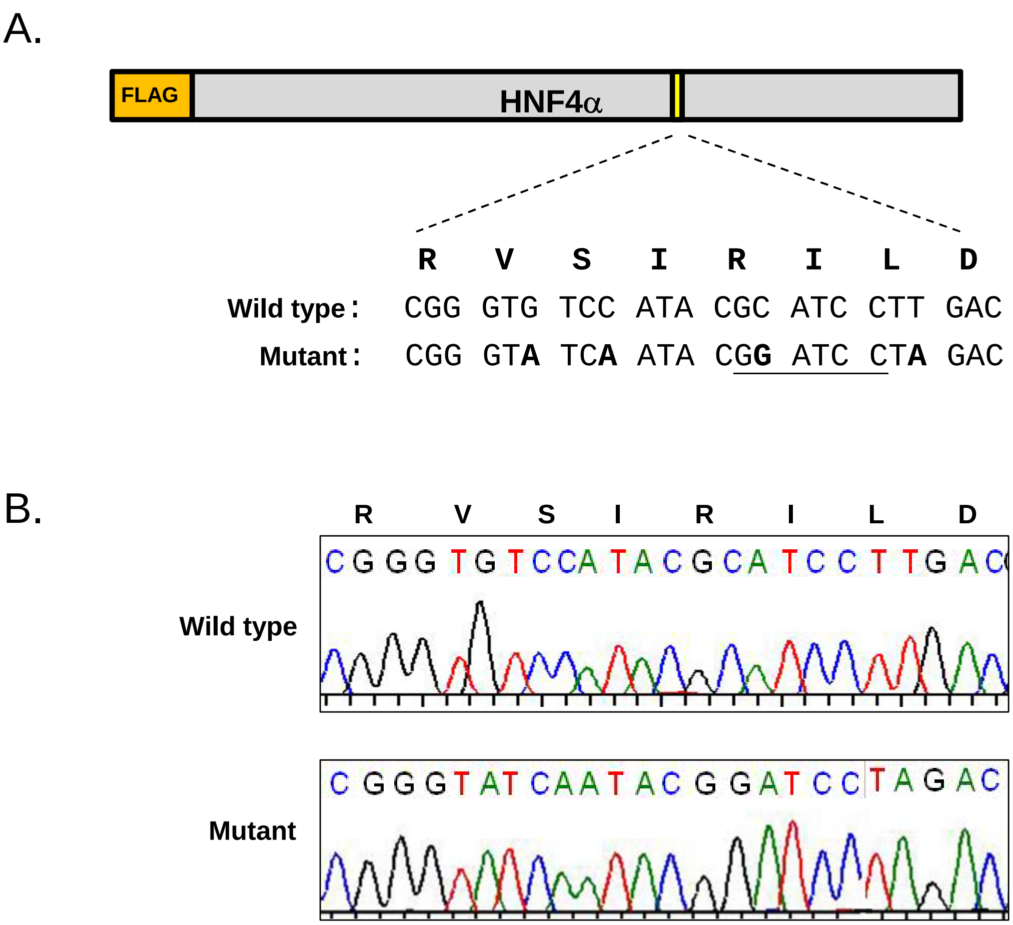

Additional file 2. Construction of the HNF4α siRNA-resistant mutant. (A). Schematic representation of FLAG-human HNF4α expression plasmid. The HNF4α siRNA target sequence (encoding amino acids 253-261) is indicated by capital letters. The silent mutations were introduced by changing the nucleotide sequences (bold letters), and a *BamHI* restriction site (underlined) was created for PCR cloning. (B). The silent mutations were confirmed by DNA sequencing.
