# Supplementary material for: Expression profile analysis of the inflammatory response regulated by hepatocyte nuclear factor 4α
Source: BMC Genomics. 2011 Feb 25;12:128. doi: 10.1186/1471-2164-12-128 (PMC3053261; doi:10.1186/1471-2164-12-128)
Supplement: Additional file 3 — siRNA rescue assay. This file shows the characterization of the HNF4α siRNA-resistant construct by Western blot, and the rescue effect of this construct on HNF4α knock-down responsive genes. [file 1471-2164-12-128-S3.PDF]

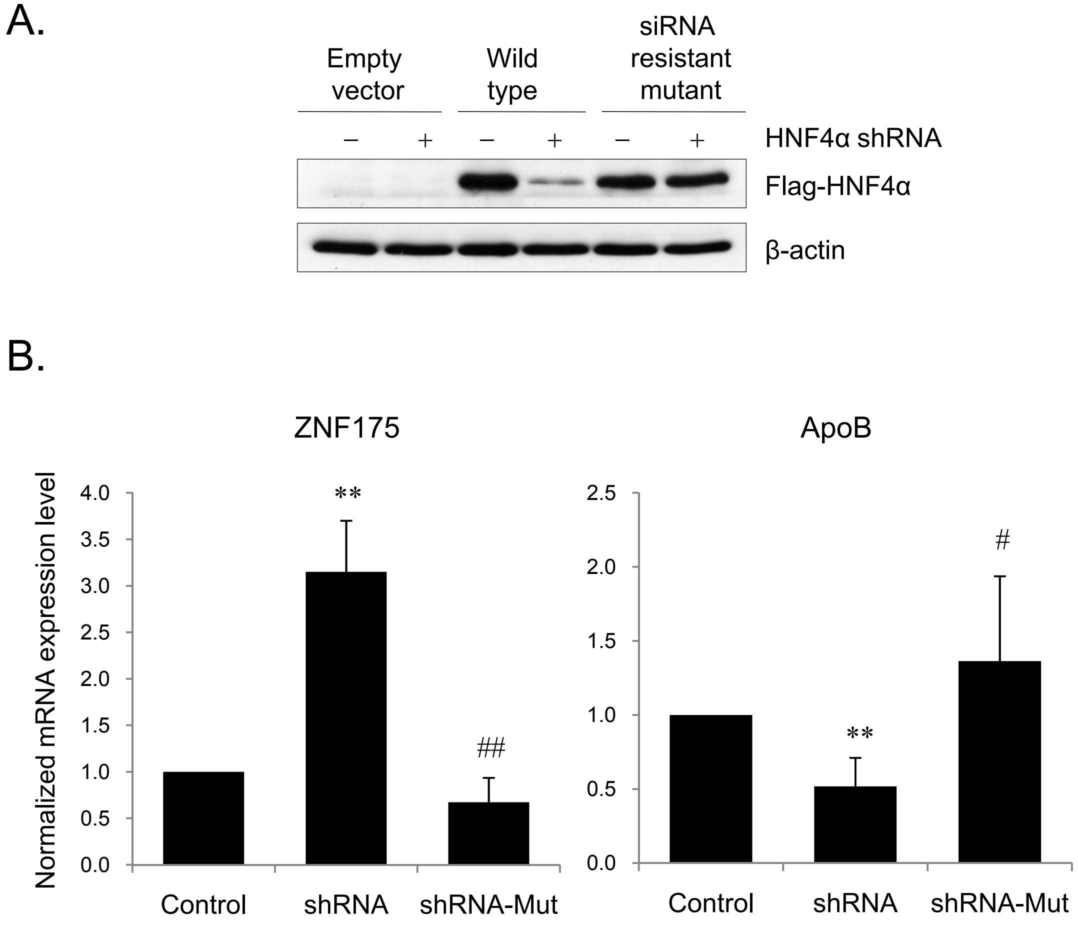

Additional file 3. siRNA rescue assay. (A). Expression of FLAG-HNF4α produced by the expression construct containing silent mutations is resistant to HNF4α shRNA. HepG2 cells were transfected with combinations of FLAG-HNF4α expression constructs (empty vector, wild type or siRNA-resistant mutant) and HNF4α shRNA. Expression level of FLAG-HNF4α protein was characterized by immunoblotting with anti-FLAG antibody. The blot was also probed using anti-β-actin antibody as a loading control. **(B).** HNF4α siRNA-resistant construct restores the siRNA-induced regulatory effect on HNF4α target genes, and the regulatory effects induced by HNF4α shRNA are consistent with our microarray data. HepG2 cells were transfected with non-specific shRNA control (Control), HNF4α shRNA plasmid (shRNA) alone or shRNA with HNF4α siRNA-resistant mutant (shRNA-Mut). The mRNA expression levels of ZNF175 and ApoB were was measured by real-time PCR. The results represent the relative mRNA expression level normalized by GAPDH mRNA level. The controls were set at 1. Data represent mean ± SD of 3 replicates. $^{**}p<0.01$ indicates a significant difference compared to Controls. $\#p<0.05$ and $\#\#p<0.01$ indicate a significant difference compared to shRNA alone.
